# A Novel Subjective Sleep Assessment Tool for Healthy Elementary School Children in Japan

**DOI:** 10.2188/jea.JE20090174

**Published:** 2010-03-05

**Authors:** Mizue Iwasaki, Akiko Iemura, Tetsuji Oyama, Toyojiro Matsuishi

**Affiliations:** 1 Japan Children’s Study Group, Research Institute of Science and Technology for Society, Japan Science and Technology Agency, Tokyo, Japan; 2 Institute for Developmental and Cognitive Neuroscience, Department of Pediatrics and Child Health, Kurume University School of Medicine, Fukuoka, Japan; 3 The Biostatistics Center, Kurume University School of Medicine, Fukuoka, Japan

**Keywords:** Japan Children’s Study Sleep Questionnaire, sleep log, school-child, sleep pattern, parental report

## Abstract

**Background:**

A child’s sleep pattern is important in defining his or her mental and physical well-being. Although we have reported previously on the utility of collecting 2 weeks of daily sleep logs, this type of record keeping is often onerous for the parents. Therefore, we established a new questionnaire, called the Japan Children’s Study Sleep Questionnaire (JCSSQ), which is used to collect sleep pattern data over 4 weeks, including weekdays, Saturdays, Sundays, and holidays.

**Methods:**

Two parent-administered sleep assessment tools, the JCSSQ and a daily sleep log, were used to examine the sleeping patterns of 105 school children (58 boys and 47 girls; age range, 6–12 years) in Fukuoka, Japan. Parents were requested to record sleep logs for 14 days after the JCSSQ. Sleep/wake status was recorded on the sleep log, from which data on the parameters of “sleep onset time”, “waking time”, “sleep period”, and “number of nights waking” were extracted.

**Results:**

There were no significant differences between the JCSSQ and the logs for waking time data collected on weekdays, Saturdays, and Sundays. However, there was a significant difference (*P* = 0.03) between the JCSSQ and the sleep logs with respect to the sleep onset time data collected on Saturdays.

**Conclusions:**

The JCSSQ was easy to fill out, and the data collected using the JCSSQ on weekdays were both valid and generally consistent with those collected using sleep logs. However, for sampling on Saturdays and Sundays, the JCSSQ data did not correlate with the sleep log data.

## INTRODUCTION

The sleep pattern of a child is an important factor in defining his or her mental, behavioral, and physical status. Therefore, precise assessment of a child’s sleep patterns is important. To evaluate the sleep patterns of children, both objective sleep assessment tools and subjective tools, such as the sleep log and questionnaire, are widely used.^1^^–^^6^

Although polysomnography has been considered the gold standard for evaluating sleep problems, its availability is restricted because it requires expensive equipment, a sleep laboratory, and a high level of expertise. In addition, it may induce anxiety in young children, thereby altering their sleep patterns. Over the past two decades, actigraphy has been developed and standardized as an objective sleep assessment tool. Actigraphs are ankle/wrist devices that continuously record limb motion over a period of weeks in the child’s natural environment.^7^^–^^9^ However, there are few actigraphic studies that address the sleep patterns of preschool children.^10^^,^^11^ Although actigraphy is less expensive than polysomnography, it is too costly to perform in studies of large cohorts.

Despite the increasing availability of objective assessment tools, subjective assessment tools, such as questionnaires and sleep logs, are still widely used. Simple questionnaires are suitable for the screening and surveillance of a large population of subjects.^12^^–^^15^ The 2-week sleep log is preferable for more detailed assessments of sleep patterns,^16^ although they impose heavy burdens both on the subjects and their parents. Recently, we reported that the results of a parent-reported 2-week sleep log correlated well with the results obtained using actigraphy.^17^ Sadeh demonstrated that the discrepancies between parental and actigraphic observations increased over a period of weeks, mainly because the parents became exhausted or less-motivated in performing such burdensome tasks over a long period.^18^

A better understanding of the characteristics of each sleep evaluation tool is urgently required. To this end, we established a new questionnaire, termed the Japan Children’s Study Sleep Questionnaire (JCSSQ), which generates representative data on three parameters over a period of 4 weeks, including weekdays, Saturdays, Sundays, and holidays, and we evaluated the utility of this new tool.

In some subjective parent-reported test systems for children, sleep onset time and waking time are addressed by a questionnaire.^8^^–^^10^ From both practical and economic perspectives, sleep-related questionnaires are useful.^11^^,^^14^

Despite the unavoidable bias associated with subjective estimations made by parents, some previous studies have revealed that sleep logs correlate well with objective estimations, even in the case of infants.^6^^,^^10^^,^^11^^,^^14^

The Japan Children’s Study (JCS) was initiated in 2005 with the following aim: “Identification of factors affecting cognitive and behavioral development of children in Japan” (based on a cohort study). The project, which was conducted in two different areas and encompassed the fields of brain science, medicine, psychology and pedagogy, was designed to clarify the development of sociality in children.^19^ To evaluate sociality in children, numerous question items were incorporated into the survey. Although sleep-related question items were considered to be important, in consideration of the burden placed on the operator, only a few such items were included. Therefore, we recently devised the JCSSQ, which is unique in that the parameters of “waking time”, “sleep onset time”, and the “number of nights waking” are addressed individually on weekdays, Saturdays, and Sundays (or holidays). In contrast, most previously reported questionnaires asked about “waking time” and “sleep-onset time”, including weekdays and weekends.^16^^,^^20^

Traditionally, Friday and Saturday nights were used to assess habitual “sleep onset time” during the weekend. Saturday and Sunday mornings were used to evaluate habitual “waking time” during the weekend, as adopted by previous studies.^10^^,^^17^

**Figure 1.**
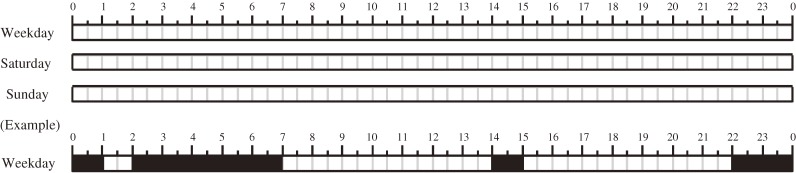
The Japan Children’s Study Sleep Questionnaire (JCSSQ). Waking time and sleep onset time were recorded separately for weekdays, Saturdays, and Sundays (or holidays). Parents were requested to complete the JCSSQ.

In the present study, we used three separate timelines to distinguish between weekdays, Saturdays, and Sundays, in a form that is simple and easy to fill in for caregivers (Figure 1).

## METHODS

### Study population

As potential volunteers, 105 children (age range, 6–12 years; 58 males and 47 females) were randomly chosen from an elementary school in Fukuoka, Japan. Introductory letters that contained information about the study were sent to the parents of these children, and subsequently (in February, 2007), the guardians of all 105 children agreed to participate in the study. This study was conducted with the guidance and approval of the Ethics Committee of Kurume University School of Medicine. Written informed consent was provided by a parent of each child participating in the study.

### Measures

#### The Japan Children’s Study Sleep Questionnaire (JCSSQ)

The JCS is a prospective cohort study of child development begun in 2005 and which is supported by the Japan Children’s Study Group, the Research Institute of Science and Technology for Society, Japan Science and Technology Agency, Tokyo, Japan. To investigate the usefulness of the JCSSQ and daily sleep logs, we scrutinized 105 volunteer school children. The sleep/wake status was recorded on the daily sleep logs, from which data on “sleep onset time”, “waking time”, “sleep period”, and the “number of nights waking” were extracted.

#### Sleep logs

Parents were requested to record a sleep log (at 30-minute intervals) over a period of 2 weeks after collection of the JCSSQ (ie, from February 2nd to February 15th, 2007). The sleep/wake status was recorded on the sleep log, from which data on “sleep onset time”, “waking time”, “sleep period”, and the “number of nights waking” were extracted.

### Statistical analysis

Data handling and all statistical analyses were carried out using the Statistical Package for the Social Sciences (SPSS) software ver. 10.0. All variables were graphically inspected to assess their distributions. The data are shown as mean (hours) ± SD (minutes). The Students *t*-test was used to compare the mean values of the groups. A *P*-value <0.05 was considered to be statistically significant. The parameters of “sleep onset time”, “waking time”, and “number of nights waking” obtained using the assessment tools were adjudged separately for weekdays, Saturdays, and Sundays (Table 1).

**Table 1.** Definitions and numbers of weekdays, Saturdays, and Sundays.

	Waking time	Sleep onset time
Weekday	Monday to Friday	Monday to Thursday^c^
	Except for second Monday^b^	Except for second Monday^b^
	9^a^	7^a^
Saturday	2^a^	2^a^
Sunday	Accounts for second Monday^b^	Accounts for second Monday^b^
(holiday)	3^a^	3^a^

^a^Indicate the number of days in each category.

^b^The second Monday was a holiday, so it was designated as a Sunday.

^c^Friday was excluded from the weekdays, considering that the next day is Saturday.

## RESULTS

We evaluated 105 volunteer school-children (58 boys and 47 girls), aged 6 to 12 years (4, 21, 28, 10, 13, 18, and 11 children with ages of 6, 7, 8, 9, 10, 11, and 12 years, respectively), who were randomly chosen from an elementary school in Fukuoka, Japan. The parents of these children recorded sleep logs over a 2-week period after collection of the JCSSQ.

### Comparisons between sleep logs and the JCSSQ

There were no significant differences with respect to waking time on weekdays, Saturdays, and Sundays (holidays) between the sleep logs and the JCSSQ. The mean waking times on weekdays were 6.51 ± 16 min and 6.46 ± 18 min in the sleep logs and the JCSSQ, respectively. The mean waking times on Saturdays and Sundays were 7.40 ± 48 min and 7.43 ± 45 min (Saturdays) and 7.46 ± 45 min and 7.42 ± 46 min (Sundays) in the sleep logs and JCSSQ, respectively. The mean sleep onset times on weekdays were 21.47 ± 34 min and 21.46 ± 33 min in the sleep logs and the JCSSQ, respectively. The mean sleep onset times on Saturdays were 22.15 ± 45 min and 22.19 ± 37 min in the sleep logs and the JCSSQ, respectively, which represented a significant difference (*P* = 0.03). The mean sleep times on Sundays were 21.52 ± 37 min and 21.49 ± 39 min in the sleep logs and the JCSSQ, respectively.

**Figure 2.**
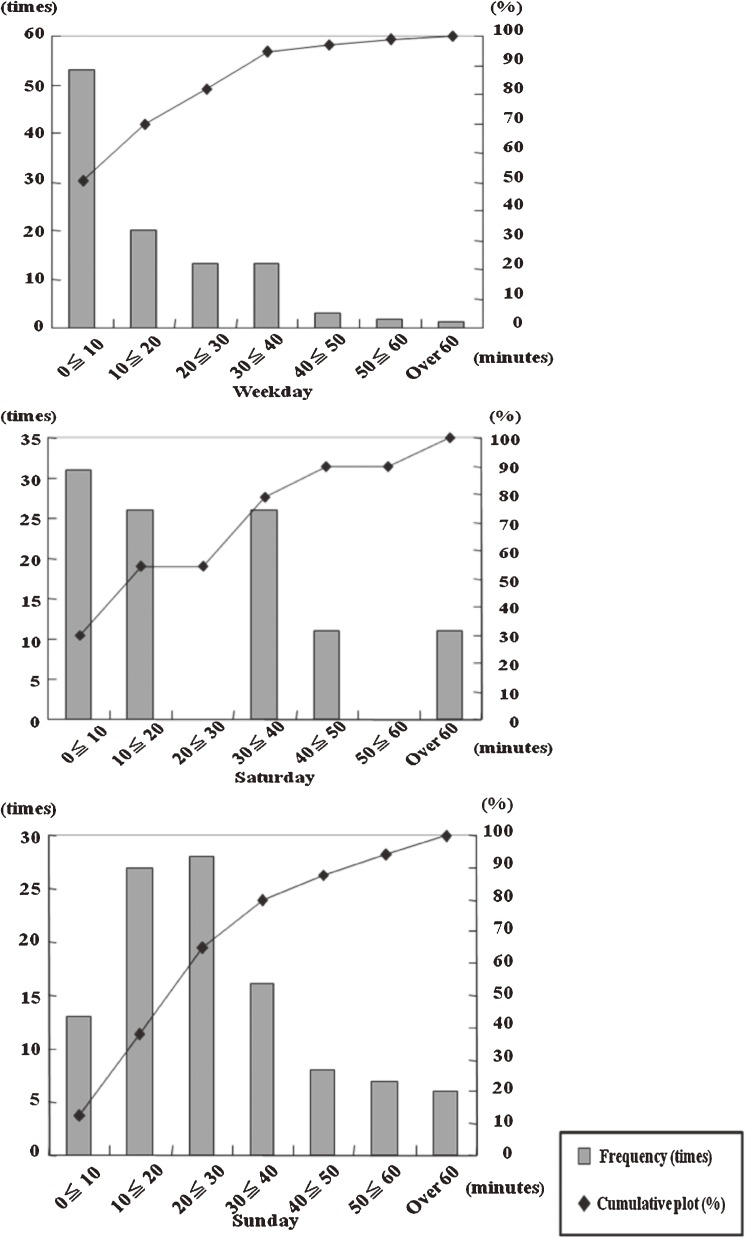
Cumulative plot and frequencies of the differences in sleep onset times. The distributions of the sleep onset times, which showed differences of less than 30 min between the sleep logs and JCSSQ, were 81.9%, 54.3%, and 64.8%, for the weekdays, Saturdays, and Sundays, respectively.

The distributions of the sleep onset times, which showed a difference of less than 30 min between the sleep logs and JCSSQ, were 81.9%, 54.3%, and 64.8% for the weekdays, Saturdays, and Sundays, respectively (Figure 2). The distributions of the waking times, which showed a difference of less than 30 min between the sleep logs and JCSSQ, were 91.4%, 41.9%, and 54.3% for the during the weekdays, Saturdays, and Sundays, respectively (Online Supplementary Figure).

## DISCUSSION

With subjective sleep assessment tools, there is a trade-off between the amount and quality of the information gathered and the burden of the participants’ tasks, which may render brief questionnaires less informative and less reliable than sleep logs.^10^^,^^11^^,^^14^ Nevertheless, sleep questionnaires are still widely used to define abbreviated sleep patterns with or without related information. The JCSSQ is a sleep evaluation tool that retains and distinguishes the data collected on weekdays, Saturdays, and Sundays (holidays) over a period of 4 weeks, while incorporating question items related to sleep onset time and awakening time.

In the present study, the distributions derived from the sleep logs and the JCSSQ, which exhibited differences of less than 30 min, were 91.4% for waking time and 81.9% for sleep onset time on weekdays, respectively. We conclude that the information on sleep patterns on weekdays provided by the JCSSQ is valid.

A previous report showed that the data from sleep logs significantly correlated with the actigram results.^9^^,^^17^ The JCSSQ obtained for a weekday may reflect the customary sleep pattern of the child. Even if the parents do not record a sleep log for the required 2 weeks, it appears that the customary weekday sleep pattern of a child is accurately evaluated by the JCSSQ. In general, the times indicated in a conventional questionnaire used to examine abbreviated sleep patterns are assumed to refer to weekday time-points. In our previous study, we reported that the questionnaire results accorded with the actigraphy on weekdays but not on weekends, which suggests that the habitual sleep pattern is usually taken to be the weekday sleeping pattern.^17^ In other words, for parents, habitual sleep is synonymous with weekday sleep.

We indicate the distribution of differences between the JCSSQ and the sleep logs for Saturdays and Sundays (holidays). There are several possible explanations for the large differences observed between Saturdays and Sundays. For example, they may due to the different study periods. In the present study, the parents were first required to fill out the JCSSQ and then record the sleep log for 2 weeks. Thus, the entry time for the JCSSQ was a Saturday or Sunday, thereby reflecting the habitual sleep pattern of each child for a Saturday or Sunday. In contrast, the sleep logs were recorded after the JCSSQ entry. Alternatively, the different patterns observed for Saturdays and Sundays between the JCSSQ and the 2-week sleep logs may be attributed to the fact that certain events, such as sports, lessons, and rest periods, are routinely scheduled over the weekend by the educators or parents of Japanese school children.

Werner and colleagues assumed that agreement between a questionnaire and sleep log would be poorer the less recent the period covered by the questionnaire, since the recorded answers could be influenced by memories, experiences, and expectations. In addition, parents often provide vague or inaccurate information, eg, stating “it depends on the situation”.^11^

### Limitations of the present study

Our preliminary studies prompted a comprehensive study to investigate the influences of family lifestyle and other environmental factors on a single study population, which is currently underway. To examine the value of the JCSSQ subjective sleep assessment tool, we used a 2-week sleep log as a reference standard. However, since a sleep log relies solely on the sleep status during weekdays and parental reports are subject to bias, the limitations of these analysis methods should be considered when comparing findings. It may also be the case that parents are unable to check rigorously the waking times and sleep onset times of their children using the JCSSQ due to the irregularity of these sleep patterns on Saturdays and Sundays; juvenile sleep patterns tend to be more irregular on weekends than on weekdays.^20^^–^^22^ The numbers of observations made in the sleep logs on Saturdays and holidays were lower than those recorded in previous reports.^2^^,^^3^ The unevenness of the distribution of the differences between the JCSSQ and the sleep logs may reflect customary sleep patterns, which may not always apply to Saturdays and holidays.

Significant biases may arise when parents fill out the forms based on memory rather than recording their observations daily, as for a sleep log. We detected these biases for both actigrams and a sleep questionnaire.^17^ In future studies, it may be important to take into account the potential biases associated with sleep-related questionnaire surveys.

### Conclusions

The JCSSQ is useful and less burdensome for parents. However, the JCSSQ generates data that are not consistent with those of the 2-week sleep logs for Saturdays and Sundays. Therefore, caution should be exercised when evaluating and interpreting sleeping pattern data collected on Saturdays and Sundays.

### Contributions of the authors

Dr. Mizue Iwasaki collected the data and evaluated the results. Dr. Akiko Iemura contributed to the enrolment of subjects’ and explained the results to the parents. Tetsuji Oyama performed the statistical analyses of the data. Dr. Toyojiro Matsuishi organized the study and contributed to discussions regarding the study.

## ONLINE ONLY MATERIALS

eMaterial.
