# Supplementary material for: A Novel Subjective Sleep Assessment Tool for Healthy Elementary School Children in Japan
Source: J Epidemiol. 2010 Mar 5;20(Suppl 2):S476–81. doi: 10.2188/jea.JE20090174 (PMC3920410; doi:10.2188/jea.JE20090174)
Supplement: eMaterial. [file je-20-S476-s001.pdf]

(Online supplementary)

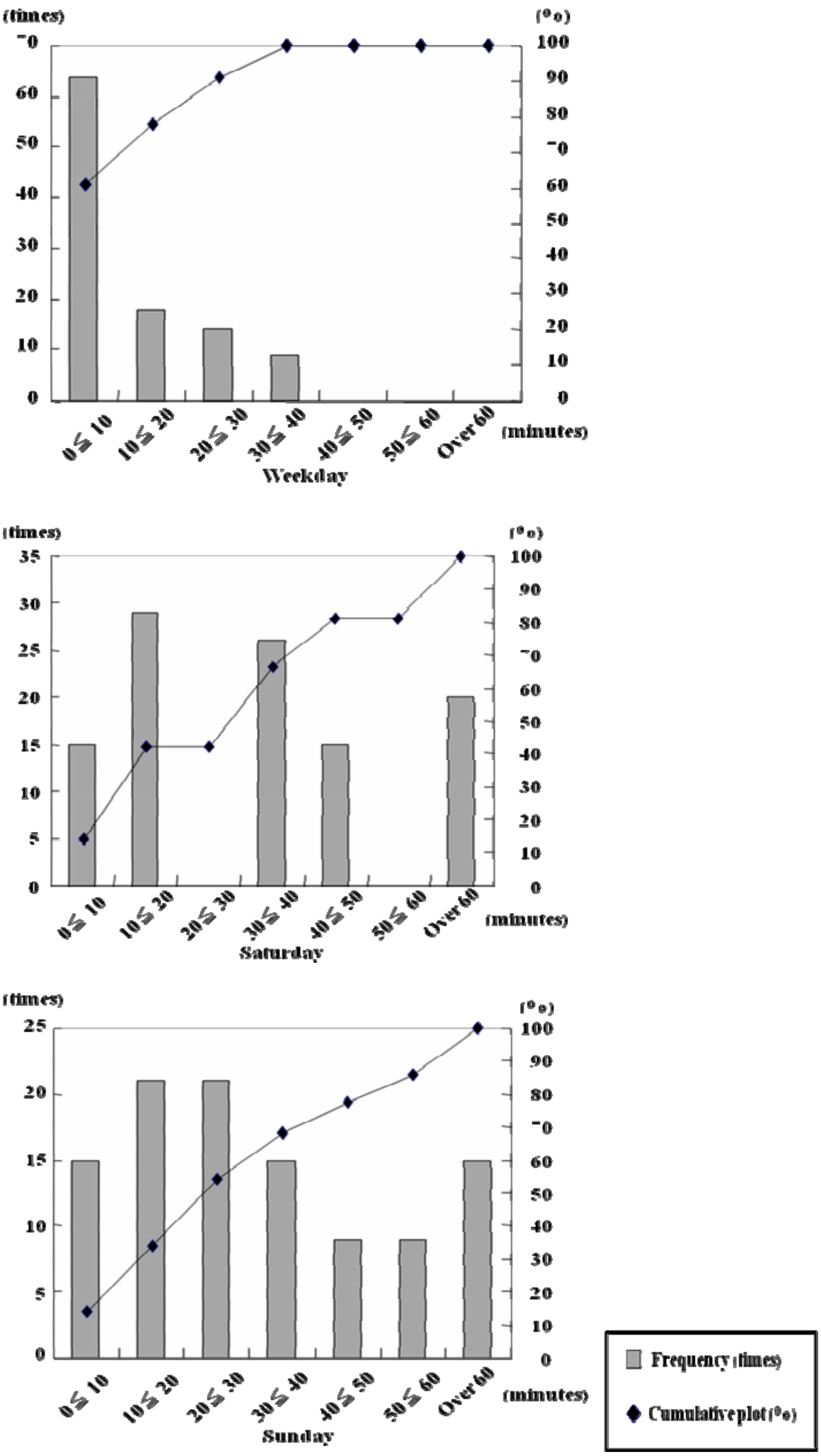

Cumulative plot and frequencies of the differences in waking time.

The distributions of the waking times, which showed differences of less than 30 min between the sleep logs and JCSSQ, were 91.4%, 41.9%, and 54.3% for the weekdays, Saturdays, and Sundays, respectively.
